# A randomized, double-blind study of the ultrasound assessment of the effect of pharyngeal packing on perioperative gastric volume in nasal surgery

**DOI:** 10.1186/s12871-019-0786-7

**Published:** 2019-07-08

**Authors:** M. Emrah Temel, Tolga Totoz, Kerem Erkalp, Gulen Safiye Temel, Aysin Selcan

**Affiliations:** 1 Tekirdag Corlu State Hospital, Corlu, Turkey; 2 Nisantasi University, Istanbul Safak Hospital, Istanbul, Turkey; 3 Health Sciences University, Istanbul Bagcılar Training and Educational Hospital, Istanbul, Turkey

**Keywords:** Pharyngeal packing, Postoperative nausea and vomiting, Ultrasonography, Gastric volume

## Abstract

**Background:**

Pharyngeal packing (PP) is commonly performed to reduce the incidence of perioperative blood ingestion (PBI) in nasal surgery (NS), and thus the incidence and severity of postoperative nausea and vomiting (PONV). This study examined the effects of PP on the perioperative gastric volume (GV) and PONV in patients undergoing NS, by ultrasound assessment.

**Methods:**

Patients undergoing elective NS [septoplasty, septo-rhinoplasty (SRP) and functional endoscopic sinus surgery (FESS)] were randomised to receive or not receive PP. In the PP group, pharyngeal packs were placed after the orotracheal intubation. Ultrasound assessments were performed for all patients preoperatively (before the anaesthesia induction) and postoperatively (before the extubation). The antero-posterior (AP) and cranio-caudal (CC) antral diameters, antral cross-sectional area (ACSA), and total GV were calculated. PONV incidence and severity were rated. These variables were compared between timepoints and groups, and in the subgroup analyses according to the surgery type. Pearson correlation analysis was performed to assess correlations between the variables.

**Results:**

AP and CC diameters and ACSAs were greater postoperatively than preoperatively in the PP and non-PP groups (*n* = 44 each; all *p* < 0.05). Postoperative AP and CC diameters and the ACSA were greater in the non-PP than in the PP group (all *p* < 0.05). Postoperative AP diameters were greater than preoperatively in patients undergoing SRP and FESS, and the postoperative CC diameter and ACSA were greater than preoperatively in patients undergoing SRP (all *p* < 0.05). Surgery duration was correlated positively with postoperative AP diameter (*r* = 0.380, *p* < 0.05), CC diameter (*r* = 0.291, *p* < 0.05), and ACSA (*r* = 0.369, *p* < 0.05). Patients who underwent septoplasty surgery, PP was decreased PONV incidence and severity at the first four hours, postoperatively (*p* < 0.05).

**Conclusions:**

The study findings indicate that PP reduces the increase in the perioperative GV due to PBI in an elective NS. It is therefore a useful and safe means of reducing the risk of perioperative pulmonary aspiration in such surgeries.

**Trial registration:**

Australian New Zealand Clinical Trials Registry (ANZCT), ACTRN12619000487112, 25/03/2019, Trial registration retrospectively registered.

## Background

The incidence of postoperative nausea and vomiting (PONV) due to perioperative blood ingestion (PBI) in nasal surgery (NS) has been observed in the 34–60% of the patients [1–3]. Blood is described as a potent emetic [4], and the incidence of PONV has been reduced with pharyngeal packing (PP) in NS [5–7]. However, a recent study indicated that there is no need to place PP to prevent PONV in NS [8], while in theory, PP may prevent PONV by preventing PBI [9].

The aim of this study was to examine the effect of PP on the perioperative gastric volume (GV) by ultrasound and our hypothesis was that the reduction of PBI with PP would reduce the PONV incidence.

## Methods

### Study design and participants

The Bagcilar Training and Research Hospital Non-Interventional Clinical Trials Ethics Committee approved this study (2016/450). All participants were informed about the study protocol and provided written consent for study participation.

With the Sample Size Calculation; 5% for error, 8% power and standard impact size: 0.53, *n* = 27 cases were found sufficiently for each group. We started the study with 120 (60 + 60) patients.

**Fig. 1:**
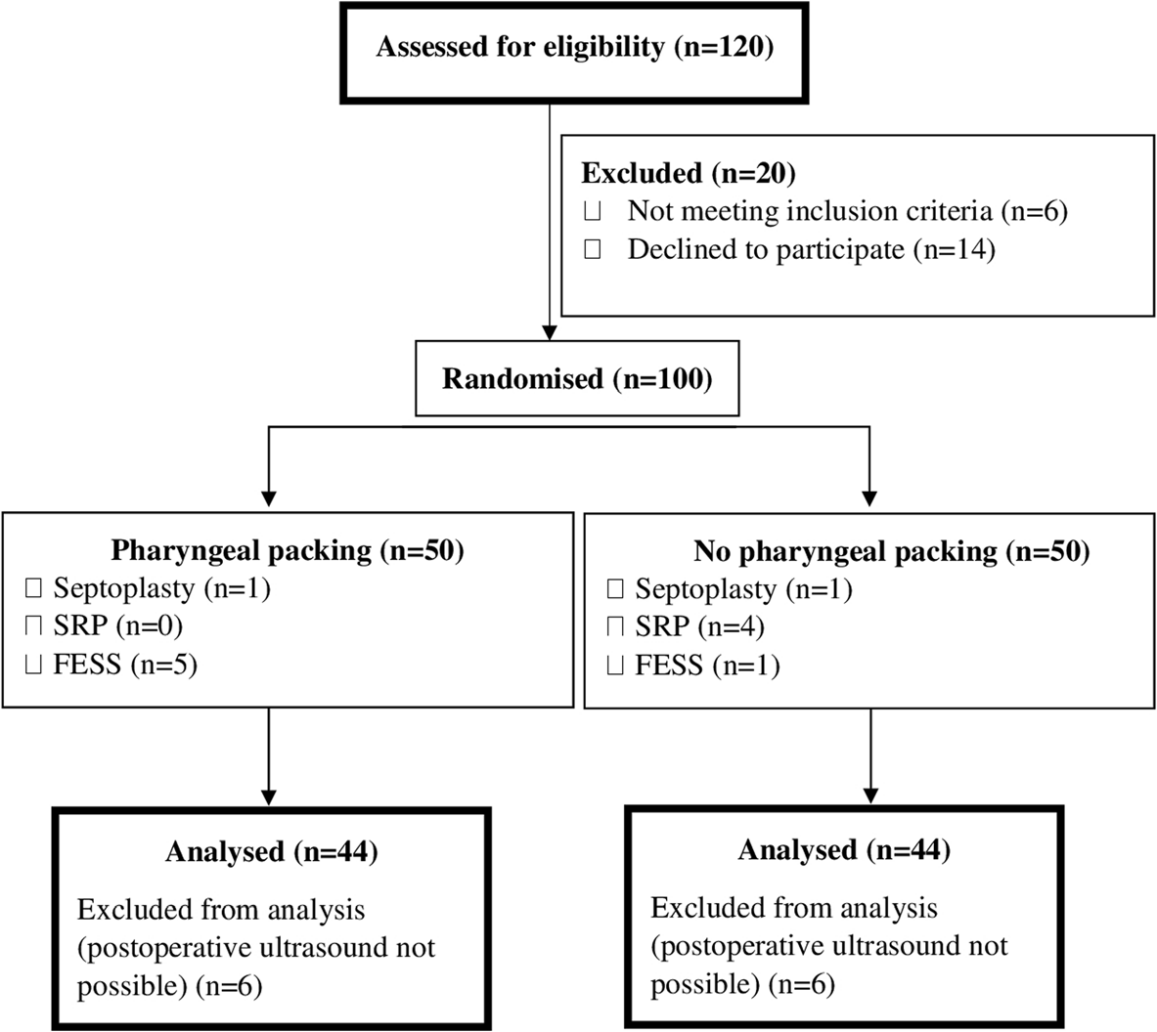
Diagram of study flow. SRP, seprorhinoplasty; FESS, functional endoscopic sinus

Patients aged > 18 years with body mass indexes < 35 kg/m^2^ and with an American Society of Anesthesiologists physical status classification of I or II, underwent an elective NS [septoplasty, septo-rhinoplasty (SRP) and functional endoscopic sinus surgery (FESS)] under general anaesthesia after an 8 h preoperative fasting. The exclusion criteria were an emergent need for surgery; risk of an increased residual GV due to pregnancy, smoking, or diabetes; upper gastrointestinal system disease; and a history of oesophageal or upper gastrointestinal surgery. The patients were randomised to receive PP (group 2) or no PP (group 1). The flow of the patient enrolment is illustrated in Fig. 1.

The study was actualized from December 2016 to June 2017.

### Procedure

Each patient was taken to the preoperative unit, and peripheral vascular access was established by a 20-G intravenous cannula. The patient was administered an intravenous infusion of 0.9% NaCl (4 ml/kg/h). None of the patients were given any analgesic or sedative drug as premedication.

A preoperative ultrasonographic examination was performed, and then each patient was anaesthetised with fentanyl (1 μg/kg), propofol (2–2.5 mg/kg) and rocuronium (0.6 mg/kg). The same anaesthesiologist performed a laryngoscopy and an intubation for all patients. Following the orotracheal intubation, soft wet pharyngeal packs were placed with gentle manoeuvres to avoid damage to the soft palate in patients in group 2. The end of the pharyngeal pack was fixed to the cheek with fastening bands such that it could be seen clearly. Patients were not administered prokinetic drugs in the perioperative period. Patients in both groups were intravenously administered ranitidine (0.5 mg/kg) following the intubation, and tenoxicam (20 mg) and tramadol (1 mg/kg) at the end of the operation for postoperative analgesia.

After the completion of the postoperative ultrasonographic evaluation, the pharyngeal packs of the patients in group 2 were removed with the gentle removal of the fastening bands. Each patient was intravenously administered 0.5 mg atropine and 1.5 mg neostigmine. Patients underwent postoperative extubation in the operating room and were taken to the postoperative care unit. After achieving a Modified Aldrete Recovery Score of 9, the patients were transferred to the ward.

### Data collection and assessments

**Fig. 2:**
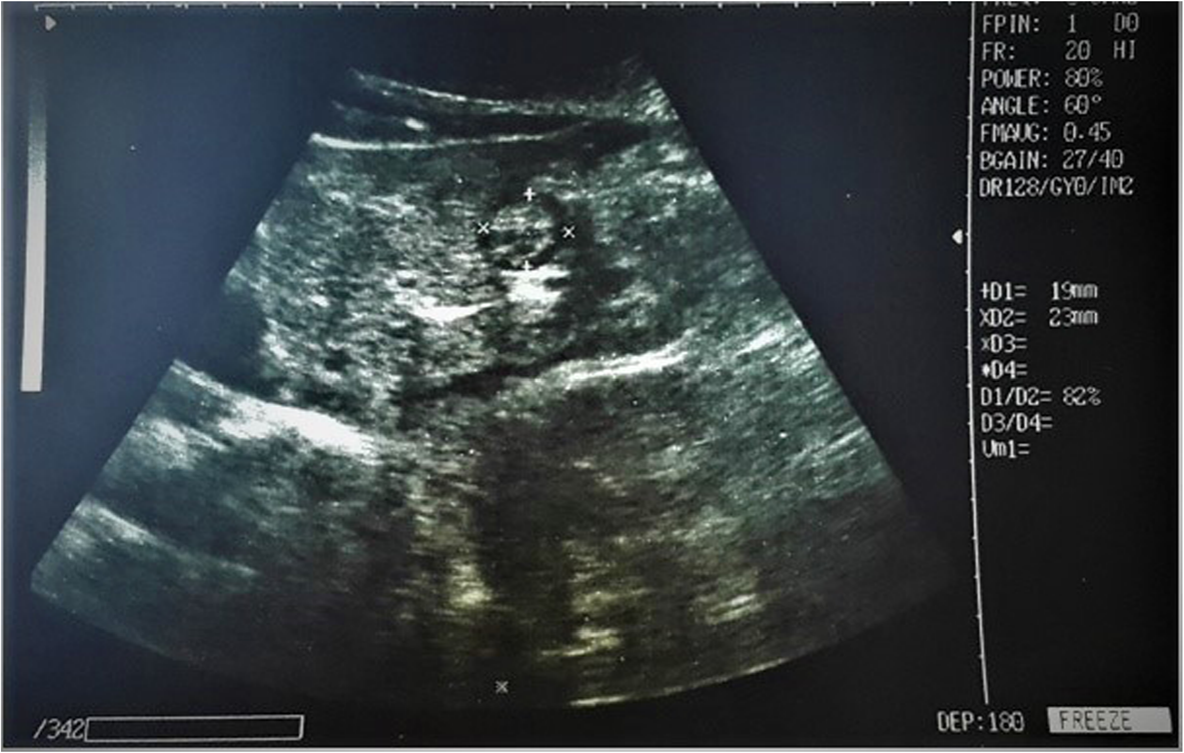
Ultrasound image of the gastric antrum (epigastric region) in the parasagittal plane, obtained after 8 h fasting in the preoperative period

**Fig. 3:**
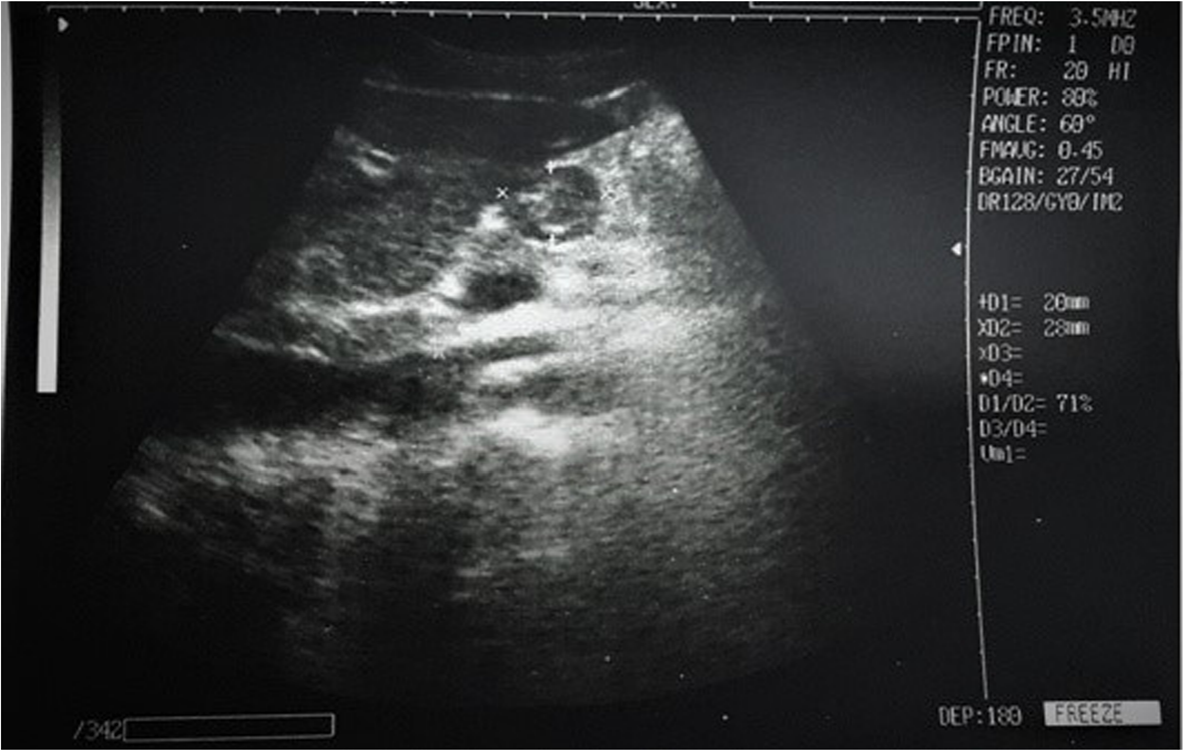
Ultrasound image of the gastric antrum (epigastric region) in the parasagittal plane, obtained in the postoperative period

The operation type and duration, and the demographic characteristics of each patient were recorded. In two separate sessions (before anaesthesia induction and before postoperative extubation; Figs. 2 and 3), an experienced radiologist with no knowledge of the group assignment and an anaesthesiologist, evaluated the stomach from the antrum by abdominal ultrasonography using a 2–5-MHz convex probe and a Shenzhen® Wed-380 system (China). Images of the stomach in a resting state were acquired between two peristaltic periods with the patient in the supine position. The postoperative examination visualised the epigastric region on the parasagittal plane. Three images were acquired from each session, and the mean antero-posterior (AP) and cranio-caudal (CC) diameters of the gastric antrum (in millimetres) were calculated. Then, the ACSA was calculated using the formula CSA = (AP × CC × 3.14) / 4 [10]. The total volume of the stomach was then estimated using a previously tested and validated mathematical model with the following formula: GV (in millilitres) = 27 + 14.6 × CSA (in square millimetres) – 1.28 × age [11].

For the comparison of PONV incidence and severity in groups 1 and 2, Kortilla’s scale [12] was used: no PONV; absence of any emetic episode and nausea, mild PONV; mild nausea or one emetic episode or short-lasting (~ 10 min) nausea of any severity triggered by an exogenous stimulus (e.g. drinking, eating or postoperative movement) followed by diminished nausea and the patient’s feeling well throughout the entire observation period with no antiemetic drug requirement, moderate PONV; one or two emetic episodes or moderate or severe nausea without exogenous stimulus or single requirement for antiemetic therapy, and severe PONV; more than two emetic or moderate to severe nauseous episodes requiring at least one antiemetic administration [13].

### Statistical analysis

Statistical analyses were conducted using the 2007 Number Cruncher Statistical System software package (UT, USA). Descriptive statistics (means and standard deviations) were calculated. For normally distributed variables, a one-way analysis of variance was used to examine the differences between time points, and Tukey’s multiple comparison test was used to examine differences between the groups. The unpaired *t* test was used to analyse differences between the groups, and the paired *t* test was used for a comparison of repeated measurements. For non-normally distributed variables, the Kruskal–Wallis test was used for the comparison between the time points and Dunn’s multiple comparison test was used to examine the differences between the subgroups. The Mann–Whitney *U* test was used to examine the differences between the groups. Qualitative data were examined using the chi-squared test, and correlations between the variables were examined using the Pearson correlation analysis. *P* values < 0.05 were considered to be significant.

## Results

Data from 88 patients (*n* = 44/group) were analysed in this study. Eight patients (four from each group) were excluded because ultrasonographic evaluation prior to extubation could not be performed due to severe gas. Four additional patients (two from each group) were excluded because postoperative gastric antrum imaging could not be performed due to the superposition of the transverse column. The demographic and surgical characteristics of the study sample are presented in Table 1.

**Table 1:** Demographic and surgical characteristics of the study groups

Characteristic	Group 1 (*n* = 44)	Group 2 (*n* = 44)	*p*
Age (years)	30.18 ± 8.98	32.36 ± 10.2	0.285
Sex			0.658
Male	29 (65.91)	27 (61.36)	
Female	15 (34.09)	17 (38.64)	
Weight (kg)	69.24 ± 12.64	75.05 ± 20.2	0.110
Height (cm)	169.34 ± 9.76	166.14 ± 17.77	0.297
BMI (kg/m^2^)	24.28 ± 4.13	25.57 ± 4.5	0.164
ASA physical status			0.368
I	31 (70.45)	27 (61.36)	
II	13 (29.55)	17 (38.64)	
Surgery duration (min)	100.91 ± 48.02	92.27 ± 39.54	0.360
Surgery type			0.251
Septoplasty	15 (34.09)	16 (36.36)	
Septo-rhinoplasty	25 (56.82)	19 (43.18)	
FESS	4 (9.09)	9 (20.45)	

Data are presented as mean ± standard deviation or *n* (%)

*BMI* body mass index, *ASA* American Society of Anesthesiologists, *FESS* functional endoscopic sinus surgery

In groups 1 and 2, AP and CC diameters, as well as ACSAs, were significantly larger in the postoperative period than in the preoperative period (all *p* < 0.05). Postoperative AP and CC diameters and ASCAs were greater in group 1 than in group 2 (all *p* < 0.05; Table 2). In patients undergoing SRP and FESS, postoperative AP diameters were greater than preoperative AP diameters (both *p* < 0.05); no significant difference in this variable was observed in patients undergoing a septoplasty. The postoperative CC diameter and ACSA were significantly greater than preoperatively in patients undergoing SRP (both *p* < 0.05), whereas no difference in these values was observed among patients undergoing a septoplasty and FESS (Table 3).

**Table 2:** Ultrasonographically obtained gastric antrum data

Variable	Group 1 (*n* = 44)	Group 2 (*n* = 44)	*p*
Pre AP diameter (mm)	14.82 ± 3.36	15.25 ± 3.72	0.569
Post AP diameter (mm)	19.34 ± 5.93	17 ± 4.76	0.044
*p*	0.0001	0.003	
Pre CC diameter (mm)	24.05 ± 4.87	21.66 ± 5.31	0.031
Post CC diameter (mm)	29.09 ± 8.37	23.45 ± 6.45	0.001
*p*	0.0001	0.032	
Pre ACSA (mm^2^)	280.06 ± 87.16	266.16 ± 109.25	0.511
Post ACSA (mm^2^)	463.17 ± 257.79	324.83 ± 155.54	0.003
*p*	0.0001	0.005	
Pre–Post AP diameter difference (%)	18.38 ± 24.48	7.56 ± 17.6	0.019
Pre–Post CC diameter difference (%)	13.39 ± 20.06	5.16 ± 19.08	0.052
Pre–Post ACSA difference (%)	27.26 ± 30.44	10.96 ± 25.16	0.007
Pre–Post ACSA difference (mm^2^)	183.11 ± 248.45	58.56 ± 131.79	0.004

Data are presented as the mean ± standard deviation

*Pre* preoperative, *AP* antero-posterior, *Post* postoperative, *CC* cranio-caudal, *ACSA* antral cross-sectional area

**Table 3:** Ultrasonographic data according to surgery type

	Septoplasty (*n* = 31)	SRP (*n* = 44)	FESS (*n* = 13)	*p*
Pre AP diameter (mm)	14.81 ± 3.87	15.23 ± 3.53	14.92 ± 2.84	0.875
Post AP diameter (mm)	15.71 ± 4.55	19.89 ± 5.95	18.23 ± 3.52	0.004
*p*	0.056	0.0001	0.016	
Pre CC diameter (mm)	22.71 ± 5.11	22.8 ± 5.12	23.38 ± 6.06	0.923
Post CC diameter (mm)	23.55 ± 6.01	28.8 ± 9.11	24.23 ± 4.85	0.01
*p*	0.133	0.0001	0.688	
Pre CSA diameter (mm^2^)	267.19 ± 102.23	274.52 ± 96.33	282.47 ± 103.81	0.890
Post CSA diameter (mm^2^)	299.22 ± 145.53	472.73 ± 260.01	353.54 ± 130.36	0.002
*p*	0.066	0.0001	0.135	

Data are presented as the mean ± standard deviation

*SRP* septo-rhinoplasty, *ESS* functional endoscopic sinus surgery, *Pre* preoperative, *AP* antero-posterior, *Post* postoperative, *CC* cranio-caudal, *CSA* cross-sectional area

Positive correlations were found between the duration of surgery and the postoperative AP diameter (*r* = 0.380, *p* < 0.05), postoperative CC diameter (*r* = 0.291, *p* < 0.05), and postoperative ACSA (*r* = 0.369, *p* < 0.05).

The incidence and severity of PONV are shown in Table 4.

**Table 4:** Incidence and severity of PONV after surgery

Surgery	Time after surgery (h)	Group 2 (*n* = 44)			Group 1 (*n* = 44)			*p*
Septoplasty (*n* = 31)	2	37.5%	Severe	15%	60%	Severe	25%	
			Moderate	15%		Moderate	15%	0.378
			Mild	7.5%		Mild	20%	
	4	18.8%	Severe	10%	60%	Severe	20%	
			Moderate	3.8%		Moderate	12%	0.046
			Mild	5%		Mild	28%	
	8	50%	Severe	10%	53.3%	Severe	8%	
			Moderate	15%		Moderate	14%	0.862
			Mild	25%		Mild	31.3%	
	24	0%	Severe	0%	13.3%	Severe	1.3%	
			Moderate	0%		Moderate	2%	0.226
			Mild	0%		Mild	10%	
SRP (*n* = 44)	2	50%	Severe	20%	50%	Severe	30%	
			Moderate	15%		Moderate	20%	0.762
			Mild	15%		Mild	10%	
	4	40%	Severe	15%	41.7%	Severe	20%	
			Moderate	10%		Moderate	11.7%	0.845
			Mild	15%		Mild	10%	
	8	25%	Severe	5%	33.3%	Severe	20%	
			Moderate	10%		Moderate	8.3%	0.786
			Mild	10%		Mild	5%	
	24	5%	Severe	0%	16.7%	Severe	6.7%	
			Moderate	0%		Moderate	5%	0.461
			Mild	5%		Mild	5%	
FESS (*n* = 13)	2	40.9%	Severe	15%	54.5%	Severe	30%	
			Moderate	20%		Moderate	14.5%	0.286
			Mild	5.9%		Mild	10%	
	4	29.5%	Severe	10%	47.7%	Severe	20%	
			Moderate	14.5%		Moderate	19.7%	0.126
			Mild	5%		Mild	5%	
	8	31.8%	Severe	16.8%	38.6%	Severe	12%	
			Moderate	12%		Moderate	14%	0.655
			Mild	3%		Mild	10.8%	
	24	2.3%	Severe	0%	15.9%	Severe	6%	
			Moderate	0%		Moderate	8%	0.064
			Mild	0%		Mild	1.9%	

*PONV* postoperative nausea and vomiting, *SRP* seprorhinoplasty, *FESS* functional endoscopic sinus surgery

## Discussion

Pharyngeal packing is commonly performed following a tracheal intubation to reduce PBI and/or tracheal contamination during ear, nose and throat (ENT), and oral surgeries. Pharyngeal packs should absorb blood and provide a physical barrier preventing PBI and reducing the incidence of PONV [6]. Despite the common belief in the efficacy of PP, it does not offer 100% protection against PBI [14]. In this study, postoperative gastric diameters and volume were increased in the postoperative period in patients who did and did not receive PP, but were greater in patients not treated with PP. Thus, patients undergoing NS who received PP ingested less blood and secretions into the stomach, than those not treated with PP. This finding is clinically meaningful in two ways. First, the perioperative ingestion of secretions and blood may result in the risk of postoperative pulmonary aspiration due to an increased GV [15–17]. Second, it can increase the incidence of PONV [18, 19]. Although some researchers have found that PP does not reduce PONV, and that it increases postoperative aphthous stomatitis and sore throat [3], many anaesthesiologists believe that PP prevents PONV by creating a physical barrier for the entry of blood from the throat into the stomach during NS [6, 15]. In our study, only patients who underwent septoplasty surgery, PP was decreased PONV incidence and severity at the first four hours, postoperatively.

Van de Putte and Perlas [20] confirmed the mathematical model for the gastroscopic and ultrasonographic evaluation of GV and published the formula used in this study for the right lateral decubitus position. According to this formula, a GV < 1.5 ml/kg reflects a low pulmonary aspiration risk [21, 22]. Arzola et al. [23] performed bedside a gastric ultrasonographic evaluation after 8 h fasting, with patients with term pregnancies in the right lateral decubitus position prior to an elective caesarean section; they calculated GVs using the above-mentioned formula. In 2011, Bouvet et al. [24] evaluated correlations between the GV and ACSA obtained by an ultrasonographic measurement with patients in the supine position. They reported preoperative ACSAs of 280 ± 115 mm^2^ and 581 ± 294 mm^2^ in elective and emergent patients, respectively, and proposed a supine ACSA cut-off value of approximately 340 mm^2^ for 0.8 ml/kg GV, which determined the aspiration risk with a sensitivity of 91% and a negative predictive value of 94%. In our study, preoperative ACSAs exceeded this cut-off in 21 of 88 (23.8%) patients. Pharygeal packing in elective NS may prevent the development of GVs carrying a postoperative pulmonary aspiration risk (threshold, 0.8 mL/kg) by 77%. However, the critical cut-off value for GV with regard to an aspiration risk must be discussed. The critical threshold for this risk has been reported to be 0.4–0.8 ml/kg (28–56 ml/70 kg) [20, 25], but the 0.8 ml/kg threshold is probably insufficient because regurgitation and aspiration of a minimum of 200 ml GV are required for pulmonary damage [26]. Residual GVs in healthy, hungry patients can be ≥1.5 ml/kg [27]. The pathophysiology of pulmonary aspiration during general anaesthesia is complex and associated with various risk factors (e.g. difficult airway management, inappropriate anaesthesia technique, straining, coughing, gastro-oesophageal reflux) [28].

There are some major limitations in this study that could be adressed in future researches. First, there were a few patients in the different nasal surgeries’ groups. Second, we did not examine the correlation between GV and PONV. Third, we did not investigate whether the duration of NS increased the GV. Fourth, we did not calculate GV using a confirmed mathematical method developed for use with angles measured in the supine position and antral CSA. Further studies are needed to evaluate the relationship between PP and perioperative pulmonary aspiration risk, and between postoperative GV and PONV. A larger sample size and different nasal surgery population need to be researched for the outcome.

## Conclusions

In conclusion, PP reduces the increase in the perioperative GV due to PBI in elective NS. It prevents the possible aspiration of blood into the aerodigestive tract. Although PP implementation is advantageous for anaesthesiologists, it seems to be disadvantageous for ENT surgeons. Cooperation is needed for this issue. Bedside ultrasound examination can aid such cooperation, enabling a determination of the degree of stomach fullness for anaesthesiologists and ENT surgeons in the operating room. Gastric volume evaluation before extubation plays a very important role, guiding decisions about gastric decompression. An increased GV is a marker of perioperative pulmonary aspiration and PONV risk. We believe that PP is a useful and safe means of reducing the risk of perioperative pulmonary aspiration in NS.

## Data Availability

The datasets used and/or analysed during the current study are available from the corresponding author upon reasonable request.

## Abbreviations

ACSA: Antral cross-sectional area
AP: Antero-posterior
CC: Cranio-caudal
ENT: Ear, nose and throat
FESS: Functional endoscopic sinus surgery
GV: Gastric volume
NS: Nasal surgery
PBI: Perioperative blood ingestion
PONV: Postoperative nausea and vomiting
PP: Pharyngeal packing
SRP: Septo-rhinoplasty
